# Effect of Progressive Balance Control Strategies on Chronic Ankle Instability in Middle-Aged Obese Women

**DOI:** 10.7759/cureus.62992

**Published:** 2024-06-23

**Authors:** Rutuja S Arulekar, Sandeep Shinde, Vrushali K Kumbhar

**Affiliations:** 1 Department of Musculoskeletal Sciences, Krishna College of Physiotherapy, Krishna Vishwa Vidyapeeth, Deemed to be University (KIMSDU), Karad, IND

**Keywords:** women, dynamic balance, fall prevention, obesity, balance strategies, ankle instability

## Abstract

Introduction: Chronic ankle instability (CAI) is a disease characterized by persistent feelings of instability in the ankle joint and a propensity for recurrent ankle sprains. It is often caused by ligamentous laxity or neuromuscular deficits. Middle-aged obese females represent a demographic subset at increased risk for CAI due to factors such as reduced proprioception and increased loading on the ankle joint. The gaps in the current evidence suggest that more research is needed on middle-aged obese females, who are particularly vulnerable to CAI due to physiological changes associated with poor balance.

Objectives: This study aims to determine the effect of progressive balance control strategies on CAI in middle-aged obese women.

Method: In this experimental study, 72 patients with CAI in middle-aged women were selected randomly using a simple random sampling method. Females aged 35-45 with a body mass index (BMI) greater than 27 kg/m2 and a history of ankle sprains greater than one and having residual symptoms. The experimental group (Group B) received progressive balance control strategies, and the conventional group (Group A) received conventional balance exercises. Foot and ankle ability measure (FAAM) scale, push-and-release test (PART), single-leg stance test (SLST), evaluations, and star excursion balance test (SEBT) were used for pre- and posttreatment.

Results: The experimental group post-intervention for static balance, dynamic balance, and postural control tests showed extremely significant improvement with a p-value of <0.0001. Between groups A and B, the dynamic balance was considered very significant, with a p-value of 0.0001. In the single-leg stance test, Group B's result was significantly greater than that of Group A's (63.4 + 16.1 and 63.4 + 16.1). PART results indicate that Group B is more significant than Group A (0.76 and 0.51, respectively).

Conclusions: The study concluded that progressive balance control strategy training is effective in middle-aged obese women with CAI.

## Introduction

Ankle instability is a condition that is associated with recurrent ankle sprains, persistent symptoms, and "giving way" that includes both functional and mechanical ankle instability [1]. Obesity is associated with impaired muscle function and postural instability, both of which increase the risk of chronic ankle instability (CAI) [2]. Ankle instability is characterized by repeated sprains, ongoing discomfort, and one that affects function. CAI, defined as persistent symptoms of instability and recurrent ankle sprains lasting longer than a year, affects about 30% of individuals [3].

People with instability have been found to have traits that differ from those of healthy individuals, yet the exact etiology is still unknown. Among these are impaired proprioception, diminished neuromuscular control, reduced range of motion (ROM), reduced strength, and altered gait. Previously, CAI was categorized as either functional instability or mechanical instability [4]. Mechanical instability can be caused by adverse anatomical changes, such as ligamentous laxity and reduced arthrokinematics [4]. Adverse neuromuscular alterations, such as reduced proprioception and neuromuscular control, may lead to functional instability [5]. Functional instability can be characterized by recurrent symptoms that do not initially appear in pathological laxity [6]. Individuals who develop CAI have mechanical or functional instability of the ankle in addition to reduced sensorimotor control and ligamentous laxity [7]. One of the most popular forms of exercise is running, which has several advantages for both physical and mental health but can also cause injuries [8]. Ankle injuries can result from running; common injuries include Achilles tendinitis and ankle sprains [9]. Pain plays a crucial physiological function by either stimulating or interfering with the motor system while simultaneously protecting the tissue from an actual or perceived threat of injury [10].

An acute ankle sprain injury must occur due to the development of CAI [11]. After an ankle injury, people with CAI frequently report episodes where their ankle feels unstable or like it could give way while doing functional tasks. Lateral ankle sprains (LASs) account for about 77%-79% of all ankle sprains [12]. Ankle sensorimotor deficits and pathological laxity are common after LAS [13]. More than 30% of patients who have had their first LAS develop into CAI even after getting professional care [14]. When an ankle sprain occurs, the most frequent mechanism of injury is twisting the ankle, which causes the ankle joint to simultaneously invert and plantarflex, stretching or partially tearing one or more ankle ligaments. The lateral ligaments of the ankle are the posterior talofibular ligament (PTL), the calcaneofibular ligament (CFL), and the anterior talofibular ligament (ATFL) [15]. The most common injury caused by an ankle sprain is to the weakest ligament, which is the ATFL [16]. A history of previous sprains gives the highest risk of inversion ankle sprains. LASs are a significant concern due to their high incidence, potential for long-term dysfunction, and high cost of medical care [17].

The ability of the lower extremities to perform movements correctly is mostly compromised by the damage that an ankle sprain causes to the ankle joint, surrounding muscles, connective tissue, and peripheral nerves [18,19]. Ankle sprains and instability are more common in obese people, suggesting that they are overweight with a high body mass index (BMI), according to the previous studies [20]. BMI is used to classify people as obese. A BMI of 30 kg/m2 or higher is considered obese [21]. Nonetheless, women have a higher probability than men of being obese. By comparison, the prevalence for men was 35.0%, and for women, it was 40.4%. The excess weight and altered biomechanics associated with obesity can lead to greater joint loading and instability during activities, predisposing obese women to ankle sprains [22]. The ankle joint is immediately impacted mechanistically by increased BMI due to the extremely high joint loading it causes. The ankle joint is susceptible to compressive loads up to eight times greater than the body weight because it carries the weight of the body. The impaired healing and rehabilitation response in obese individuals can contribute to the development of CAI [23]. Greater BMI is associated with poor balance control, postural instability, and mobility problems. Obese people may be more likely to increase their risk of falling [24]. This training on balance control strategies focuses on automatic postural responses in addition to static and dynamic balance management. Proper coordination between the afferent (sensory) information passed and the efferent (motor) response to contract an appropriate muscle pattern for maintaining and achieving the state of balance during all functional activities is hampered [25].

This will facilitate the central nervous system to retrain the interpretation of somatosensory input, which is necessary for maintaining balance, preventing falls, and preventing recurrent sprains. It will also improve the body's response and muscle activation pattern [26]. This study addresses progressive balance control strategies and outcome measures. The researcher wants to find the effect of progressive balance control strategies on CAI in obese middle-aged females.

## Materials and methods

Study design

This experimental study has been carried out in Krishna Vishwa Vidyapeeth, Karad, after receiving approval from the Institutional Ethical Committee from August 2022 to May 2023. Participants’ permission to be in the experiment was obtained by signing the consent form. A total of 72 individuals fulfilled the inclusion criteria and participated actively in the study. Then, those 72 patients were randomly allocated into two groups, namely, Group A which is the control group includes 36 patients, and Group B which is the experimental group includes 36 patients, by simple random sampling method. The study duration was 12 months at Krishna Hospital, Karad, in the physiotherapy outpatient department. The intervention was done for six weeks. Patients who are females ranging in age from 35 to 45 years old, participants with unilateral ankle instability, a BMI greater than 27 kg/m^2^, and a history of more than one ankle sprain and residual symptoms were included. Subjects were excluded if they had a history of neurological impairment or vestibular disorder; previous lower limb surgeries; any known vision deficit other than myopia, hyperopia, or astigmatism; and were on sedatives, antiepileptics, and antidepressants. The diagnosis of ankle instability was based on symptoms and physical examination and supported by a foot and ankle ability measure. The included ankle was assessed before and after treatment for pain and functional assessment scales and tests.

Sample size calculation

G-Power 3.1 software was the tool used to evaluate the sample size. A total of 72 participants participated in this research and were randomly allocated into two groups by the random sampling method, i.e., Group A includes 36 patients and Group B includes 36 patients. Participants in Group A received conventional balance treatment, and participants in Group B received progressive balance control strategy training.

Procedure

All the patients were explained about the study procedure, the intervention, and the benefits of the current research work, along with the written consent and verbal informed consent that were obtained from all the patients before being included in this study. Prior to the procedure, a written and informed consent form was signed. The purpose and methods of the research were explained to the participants. The single-leg stance test (SLST), star experiment balance test (SEBT), push-and-release test (PART), and foot and ankle ability measure (FAAM) scale were used for the pre- and post-test assessments for both groups.

Intervention

The six-week intervention program, progressive balance training, consists of 18 supervised sessions. There were three sessions a week, lasting 45 minutes each. Most exercise was performed in the parallel bar to gain patients' confidence, and appropriate rest time was given. Ultrasounds for eight minutes x 0.8 cm^2^ were given for the first two weeks before every session. A hot moist pack for 15 minutes was utilized on the ankle joint after every treatment session. Subjects were advised to consult a dietician for obesity management. For both groups, this is the baseline course of treatment.

For Group A, the exercises included ankle isometrics, chair exercises, lateral bending, and spot marching while seated. Participants also performed exercises such as standing in a double-leg stance with open eyes on a solid surface, standing in a double-leg posture with closed eyes on a solid surface, spot marching, adopting a double-leg stance with open eyes on an uneven surface, standing in a single-leg posture with open eyes on a sturdy surface, maintaining a tandem stance with open eyes on a solid surface, standing in a tandem stance on uneven terrain, standing in an SLST with closed eyes, and walking in tandem [27,28].

For Group B, all of the control group's exercises were included, along with the double-leg stance and open-eye perturbations: adopting a double-leg position and grasping a medicine ball, posing with both legs crossed and bending over, standing on one leg while bending over, stepping with crossed, or braided legs or stepping in tandem while experiencing disturbances [29-31].

Outcome measures

SLST

This is an assessment test used to assess static balance. This test uses a single-leg stance on a firm surface with open eyes and hands on the hip. Interrater reliability is 0.89, and this test is highly valid [32,33].

SEBT

This is an assessment test for dynamic postural control consisting of a series of lower-extremity-reaching tasks in different directions. The intraclass correlation coefficient is from 0.78 to 0.96 [34,35].

PART

This is a highly valid measure to check the response of postural control with an intraclass correlation coefficient of 0.84 [36,37].

FAAM

FAAM is a self-report measure that assesses the physical function of individuals with lower leg, foot, and ankle musculoskeletal disorders. Interrater reliability is 0.87 [38].

Statistical analysis

The IBM SPSS Statistics for Windows, Version 26.0 (Released 2019; IBM Corp., Armonk, New York, United States) was utilized for all statistical analyses. For each group, the quantitative variables' means and standard deviations were computed. The difference in mean scores between the pre- and post-measurements for each group was ascertained using a paired t-test.

In all statistical tests, a p-value <0.05 was considered significant. The descriptive data were expressed as mean, standard deviation, and percentages for nominal variables using the chi-square test.

## Results

This study was carried out among 72 females with CAI. According to statistical analysis, the effect of progressive balance strategies was significantly effective for CAI as compared to conventional exercise program. There was a significant reduction in the SLST, SEBT, PART, and FAAM scale. 

Demographic variables

Table 1 shows the comparison of patients' demographic characteristics in both groups. Moreover, it also shows the baseline demographic and clinical characteristics of the patients.

**Table 1:** Demographic variables BMI: Body mass index

Variables	Mean (SD)	
	Group A (control group)	Group B (experimental group)
Age (years)	41.222 (3.424)	39.722 (3.309)
Height (cm)	172.66 (9.125)	171.18 (9.428)
Weight (kg)	74.472 (12.896)	75.108 (13.723)
BMI (kg/m^2^)	27.694 (1.465)	27.95 (1.659)

SLST

Table 2 shows that Group B's SLST score was significantly higher than that in group Group A (63.4 and 67.9, respectively). Both groups were found to be statistically extremely significant with p < 0.0001.

**Table 2:** Comparison of pre- and posttest mean scores of SLST within Group A (control group) and Group B (experimental group) SLST: Single-leg stance test

	Pretest	Posttest	Mean difference	p-value
Group A	11.9 ± 3.5	63.4 ± 16.1	51.5	<0.0001
Group B	12.1 ± 3.3	67.9 ± 15.4	55.8	<0.0001

SEBT

In Table 3, in the anterior direction, both Group A and Group B showed improvements in reach distance from pretest to posttest. However, Group B demonstrated a greater mean difference (8.2) compared to Group A (4.1) indicating that the intervention was more effective in enhancing anterior reach distance in Group B. In the anterolateral side, both groups exhibited improvements, but Group B showed a significantly higher mean difference (14.0) compared to Group A (3.9). For the anteromedial side, Group B again displayed a greater mean difference (8.6) compared to Group A (5.2), indicating that the intervention was more effective in enhancing anteromedial reach distance in Group B.

**Table 3:** Comparison of SEBT mean scores between groups A (control group) and B (experimental group) before and after the test SEBT: Star excursion balance test

	Pretest	Posttest	Mean difference	p-value
Anterior				
Group A	53.2 ± 3.3	57.4 ± 3.3	4.1	<0.0001
Group B	52.9 ± 7.1	61.2 ± 7.6	8.2	<0.0001
Anterolateral				
Group A	43.4 ± 4	47.3 ± 4.1	3.9	<0.0001
Group B	46.4 ± 7.3	60.4 ± 7.1	14.0	<0.0001
Anteromedial				
Group A	52.4 ± 4.5	57.6 ± 4.5	5.2	<0.0001
Group B	53.4 ± 7.3	62 ± 7.5	8.6	<0.0001
Posterior				
Group A	42.5 ± 5.1	46.1 ± 5.2	3.6	<0.0001
Group B	62.9 ± 7.5	70.3 ± 7.8	7.4	<0.0001
Posterolateral				
Group A	29.6 ± 5.6	34.8 ± 6.1	5.1	<0.0001
Group B	36.6 ± 7.3	50.6 ± 7.2	14.0	<0.0001
Posteromedial				
Group A	41.4 ± 5.9	44.3 ± 6.1	2.8	<0.0001
Group B	51 ± 7.4	58.2 ± 7.5	7.1	<0.0001
Medial				
Group A	51.2 ± 5.7	54.7 ± 5.9	3.5	<0.0001
Group B	53.5 ± 7.6	60.2 ± 7.6	6.6	<0.0001
Lateral				
Group A	34.1 ± 4.2	37.1 ± 4.3	3.0	<0.0001
Group B	34.3 ± 7.8	42.4 ± 8.2	8.0	<0.0001

In all these, posterior, posterolateral, and posteromedial directions, Group B consistently showed substantially higher mean differences compared to Group A, indicating that the intervention had a more significant impact on these directions for Group B. In medial and lateral directions, Group B showed improvement than in Group A in both medial and lateral directions as well, with higher mean differences, indicating significant improvement in reach distance for both directions due to the intervention.

PART

Table 4 shows the results obtained before and after the intervention for pre- and posttest values for PART. There was statistically significant improvement in post PART score of Group B than Group A (0.76 and 0.51, respectively).

**Table 4:** Comparison of the PART's pre- and posttest mean scores within Group A (control group) and Group B (experimental group) PART: Push-and-release test

	Pretest	Posttest	p-value
Group A	1.59 ± 0.7	0.76 ± 0.6	<0.0001
Group B	1.36 ± 0.4	0.51 ± 0.5	<0.0001

FAAM

Table 5 shows the pretest and posttest values for foot and ankle ability measure for both the Group A and Group B. Both groups were found to be statistically extremely significant with p < 0.0001.

**Table 5:** Comparison of the FAAM scale mean scores for Group A (control group) and B (experimental group) before and after the test FAAM: Foot and ankle ability measure

	Pretest	Posttest	p-value
Group A	46.81 ± 5.9	61.71 ± 6.6	<0.0001
Group B	47.11 ± 6.2	71.88 ± 5.5	<0.0001

## Discussion

CAI damages the mechanoreceptors located within the lateral ankle ligaments and has been shown to negatively alter proprioceptive and postural control, which helps an individual prevent falls [39].

The purpose of the present study, "Effect of progressive balance control strategies on chronic ankle instability in middle-aged obese females," was to ascertain the effect of progressive balance strategies as well as to compare their effectiveness with traditional balance control treatments on CAI in middle-aged obese females.

Using outcome measures such as the PART to evaluate postural control response, the SLST to evaluate static balance control, and the SEBT to evaluate dynamic postural control. Those tests are more reliable and valid. FAAM is a self-report measure that assesses the physical function of individuals with lower leg, foot, and ankle musculoskeletal disorders, which was also used in the study. As a result of this study, both groups' performance on physical functional tasks, postural response, and balance all significantly improved. When comparing the two groups, it was proven that dynamic balance and postural control were improved by the conventional balance training program in middle-aged, obese females with CAI. The progressive balance control exercises, which focused on balance control strategies, improved static balance, dynamic balance, and automatic postural reactions. 

According to a study by Baldwin, people with a higher BMI have more chances of suffering from ankle sprains because their BMI directly affects their ankle joints due to abnormally high joint loading. This is primarily because women's physiological changes are greater than those of men, making them more susceptible to injury. In addition, women experience CAI 2.6 times more frequently than men [39]. Our study supports Baldwin's findings regarding the impact of BMI and gender on ankle instability while extending the discussion to emphasize the effectiveness of progressive balance control strategies in mitigating these risks.

In a breakthrough study on CAI and aging, Kosik observed that studies on CAI have focused on young adults; middle-aged and older adults with a previous history of ankle sprains have received less attention [40]. Blenkinsop conducted a study on 12 gymnasts to find out which control strategies worked the most. A total of 12 gymnasts participated in a study by Blenkinsop to evaluate the most effective control techniques [41]. It was discovered that multiple approaches can coexist at one moment; only one or two may dominate throughout a work. The results of this research indicate that postural control can be enhanced by including exercises in rehabilitation programs that specifically target these control methods and activate the right muscle pattern. As a result, as anticipated, the exercises in our study improved the muscle groups' coordinated actions or balance control techniques [41]. This also restricts the knowledge of whether current treatment techniques work in these age groups. In this study, we have taken middle-aged obese females and focused on progressive balance control strategies to see whether this tailor-made protocol is effective in this age group or not. The study's findings show a significant improvement in the middle-aged, obese females' postural control.

McKeon and Hertel carried out a study on 31 young participants with CAI, where outcome measures like the SLST and the test for star excursion balance were used to assess static and dynamic balance. Four weeks of progressive balance training were given to one group, and the other group was the control group. The findings of this research showed that the conventional balance training plan, which focused more on dynamic reaching tasks and dynamic stabilization after disturbances, was less beneficial than progressive balance training [42]. Our study corroborates McKeon and Hertel's findings by demonstrating the superior efficacy of progressive balance training over conventional methods in improving balance and postural control in middle-aged obese females with CAI. By integrating these insights into clinical practice, clinicians can advance rehabilitation strategies and optimize outcomes for individuals managing CAI.

Simpson et al. carried out a study whose purpose was to examine neuromuscular control and ankle kinematics in individuals with CAI. The findings from the study indicated alterations to neuromuscular control and ankle kinematics in individuals with CAI. This research recommends further examination of the potential anticipatory and reactive motor control strategies in individuals with CAI, which would assist clinicians and researchers in developing effective training programs intended to increase the control strategies in CAI [43]. Our study contributes to the evolving understanding of CAI rehabilitation by demonstrating the efficacy of progressive balance control strategies in improving balance and postural stability in middle-aged obese females.

Our study focused on all three domains of balance control, i.e., static balance, dynamic balance, and autonomic postural reaction. According to recent advances, the new outcome measure, the PART, was used in this study, which is a novel approach to assessing postural control. The balance control technique training program used in this study was found to effectively treat all three of the balance control domains. The results of the current study align with and extend previous research on CAI in obese women. Consistent with prior studies, our findings show that obese women with CAI exhibit significant deficits in postural control compared to healthy controls. Specifically, we found that the obese CAI patients required more time to stabilize after performing a task like jumping, indicating impaired neuromuscular control [44].

The results of this study, which show an improvement in obese middle-aged women with CAI, corroborate previous findings and suggest that management of balance strategies has been performed to increase the study population's postural stability. However, our study uniquely demonstrates that a novel balance control technique training program was effective in improving postural stability in this population. This contrasts with previous research that has primarily focused on conventional ankle strengthening and proprioceptive exercises, which have shown limited effectiveness for obese individuals with CAI [45].

So, this study provides us with a greater insight that progressive balance control strategies are more beneficial and have a more clinically effective impact on balance and postural control than the conventional balance exercise program in middle-aged obese women with CAI.

Limitations

There are several limitations to this study. Firstly, the research was confined to one geographical location, potentially limiting the applicability of the findings to other settings. Secondly, due to constraints in resources and time, the study did not encompass samples from the obese class 3 category, which might have offered insights on the intervention's efficacy across different weight categories. Lastly, the study sample exhibited heterogeneity in terms of occupation, which might have introduced variability in response to the intervention and influenced the study outcomes.

Future recommendations

Future research has the aim of broadening the analysis' geographic reach to include multiple locations or regions. This would enhance awareness of the intervention's effectiveness across different demographic and cultural contexts. To obtain a more holistic comprehension of the intervention's impact, future studies should prioritize the inclusion of samples from the obese class 3 category. This subgroup often presents unique challenges and may respond differently to interventions compared to other weight categories. Extending the duration of the treatment period or changing the study design to suit this demographic may provide crucial information about how to tailor the exercise protocol to individuals who are extremely obese. The future research should include participants based on different occupations in the same age group. Conducting studies with extended follow-up periods would provide a more robust assessment of the long-term effects of the intervention and track any sustained effects of the intervention strategies.

## Conclusions

This study concluded that when the two exercise programs were compared, progressive balance control strategies with conventional exercise program results were more significant than conventional exercises. Hence, progressive balance control strategies have a more clinically beneficial impact on postural control than conventional balance exercise programs on CAI in middle-aged obese females. The findings showed more improvement in dynamic balance, static balance, postural response, and functional performance of the ankle joint in an individual.
